# Biosynthesis of a Novel Ginsenoside with High Anticancer Activity by Recombinant UDP-Glycosyltransferase and Characterization of Its Biological Properties

**DOI:** 10.3390/molecules30040898

**Published:** 2025-02-14

**Authors:** Dandan Wang, Yan Jin, Hongtao Wang, Chenwei Zhang, Yao Li, Sathiyamoorthy Subramaniyam, Jae-Kyung Sohng, Nam-In Baek, Yeon-Ju Kim

**Affiliations:** 1 College of Life Sciences, Yantai University, Yantai 264005, China; whdandan@163.com (D.W.); ytuwht@163.com (H.W.); zchenwei0620@163.com (C.Z.); liyao723@163.com (Y.L.); 2 School of Life Science, Nantong University, Nantong 226019, China; jinyan224@hotmail.com; 3 Research and Development Center, Insilicogen Inc., Yongin 16954, Gyeonggi-do, Republic of Korea; 4 Department of Pharmaceutical Engineering, Institute of Biomolecule Reconstruction, SunMoon University, Asan-si 31460, Chungnam, Republic of Korea; sohng@sunmoon.ac.kr; 5 Graduate School of Biotechnology, College of Life Science, Kyung Hee University, Yongin-si 17104, Gyeonggi-do, Republic of Korea; nibaek@khu.ac.kr

**Keywords:** *Salinispora tropica*, sterol glucosyltransferases, ginsenoside Rh2, novel ginsenoside, molecular docking

## Abstract

UDP-glycosyltransferases (UGTs) contribute to catalyzing the glycosylation of numerous functional natural products and novel derivatives with improved bioactivities. UDP-glucose sterol glucosyltransferase (SGT) is normally involved in the synthesis of sterol glycosides in a variety of organisms. SGT was derived from *Salinispora tropica* CNB-440 and heterologously expressed in *Escherichia coli* BL21 (DE3). Novel 12-*O*-glucosylginsenoside Rh2 was identified using HPLC, high-resolution MS (HR-MS), and NMR analysis. The cell viability assay was performed on 12-*O*-glucosylginsenoside-treated AGS stomach cancer, HeLa cervical cancer, U87MG glioma, and B16F10 melanoma cell lines. Protein structure modeling, molecular docking, and dynamics simulations were performed using AutoDock 4.2 and GROMACS 2020.1 software. The SGT gene is comprised of 1284 nucleotides and codes for 427 amino acids. The 12-*O*-glucosylginsenoside Rh2 may be a potential anticancer agent due to its potent viability inhibition of cancer cells. Structural analysis showed critical perspectives into the intermolecular interactions, stability, and binding energetics of the enzyme–ligand complex, with outcomes complementing the experimental data, thereby deepening our understanding of the structural basis of SGT-mediated glycosylation and its functional implications. This report presents a novel ginsenoside, 12-*O*-glucosylginsenoside Rh2, utilizing reshuffled SGT derived from *S. tropica*, and provides a promising candidate for anticancer drug research and development.

## 1. Introduction

Ginseng, scientifically known as Panax ginseng Meyer, has a long history of usage throughout Asia, dating back thousands of years, both as an important component of traditional medicine and as a nutritional supplement [1]. It is also well-known as a tonic traditional medicinal plant around the world. Ginsenosides are significant pharmacologically bioactive ingredients in ginseng [2]. Naturally occurring ginsenosides in wild, uncultivated ginseng have been identified as part of the ginseng’s composition [3]. They can be divided into groups called protopanaxadiol (PPD) and protopanaxatriol (PPT) and further glycosylated by glucose and other sugars, producing a variety of ginsenosides [4]. Different ginsenosides have been reported to exert different pharmacological effects [5]. Various biological functions are exhibited by ginsenosides, including immunity-boosting [6], anticancer [7], anti-inflammatory [8,9], anti-oxidative, and anti-aging effects. Moreover, studies suggest that a characteristic component of red ginseng with a dammarane skeleton, called ginsenoside Rh2, has various potential bioactivities [10] and can be an anticancer agent [7,11,12,13,14,15,16]. Since 2006, China has approved ginseng as a food supplement [16]. Enzymatic biotransformation is a specific and selective process that does not pollute the environment, as compared to the physical and chemical transformation of ginsenosides. Therefore, it may be considered the most promising technique due to its high selectivity and productivity.

The transfer of active sugar donors to different acceptor molecules is catalyzed by enzymes known as glycosyltransferases (GTs). Glycosylation is one technique that plants use to modulate the bioactivity of lipophilic substances and, as a result, cellular homeostasis [17]. In plants, glycosylation is essential for chemical transformation, signal detection, and secondary metabolism, which could increase water solubility and improve natural product absorbability. Sterol glucosyltransferases (SGTs) convert the glucose molecule into sterols and related compounds, producing glycosyl derivatives and steryl glycosides with various pharmacological and biological processes [18]. SGTs are classified into families based on a scheme that currently describes over 90 GT types (http://www.cazy.org/, accessed on 10 January 2025). Key enzymes that catalyze the critical last stage of ginsenoside production are related glycosyltransferases. As previously reported, glycosyltransferases play a crucial role in modifying ginsenoside aglycones, thereby leading to the generation of complex and varied ginsenosides with significant pharmacological effects [14]. Pharmacological investigations have demonstrated that extensive glycosylation modification is the fundamental cause of the formation of a wide variety of ginsenoside types. Each type of ginsenoside exhibits distinct pharmacological effects, effects which can be completely opposite. Thus, ginsenoside aglycone glycosylation has been extensively researched more recently (Table 1).

This research involved the successful cloning of the SGT gene from *Salinispora tropica* CNB-440 and its heterologous expression in *E. coli* BL21 (DE3) and the production of a novel glycoside derivative. The anticancer properties of the novel and parental derivatives were assessed with relevance to ginsenoside Rh2 in AGS, B16F10, HeLa, and U87MG cells. The cytotoxicity assay results demonstrated that the novel ginsenosides displayed remarkable inhibition effects on cancers cell lines at a concentration of 50 μM. In contrast, when compared with Rh2, the novel ginsenoside showed negligible toxicity in normal RAW264.7 cells. These findings suggest that the new derivative could be regarded as a potential pharmaceutical candidate for the treatment of various cancer diseases in future research. Structural analysis demonstrated that ginsenoside Rh2 formed two hydrogen-bonding interactions with GLN314 and GLN245. Furthermore, the residues PHE42, TRP244, PRO247, and PRO316 participated in hydrophobic interactions. The selected ligand of recombinant SGT had the potential to generate stable uridine—5′-diphosphate—glucose (UDPG)-ginsenoside Rh2 complexes, which may have implications for further research on bioactivities related on ginsenosides.

## 2. Results

### 2.1. Heterologous Expression and Purification of SGT

**Figure 1:**
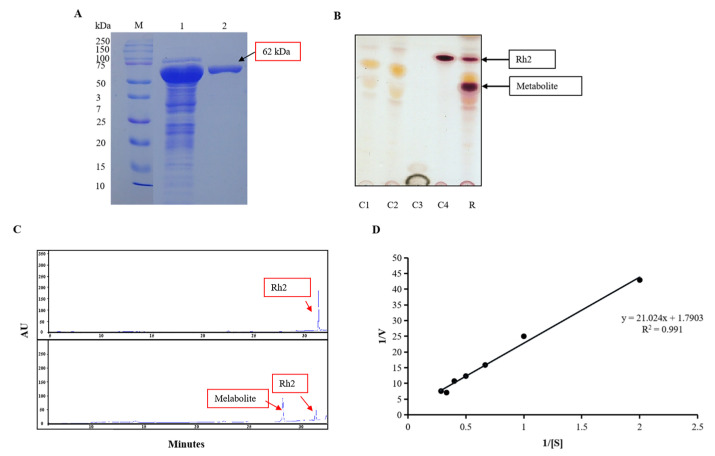
Biotransformation of metabolite. (**A**) SDS-PAGE analysis of recombinant SGT; M: marker (10–250 kDa, Ppromega); 1: crude enzyme; 2: purified enzyme. (**B**) TLC analysis of transglycosylated products synthesized from ginsenoside Rh2 and SGT crude enzyme; C1: control 1-enzyme; C2: control 2-enzyme and UDP-glucose; C3: UDP-glucose; C4: ginsenoside Rh2 and UDP-glucose; R: result of synthesis. (**C**) HPLC analysis of transformation time course of ginsenoside Rh2 by recombinant SGT. (**D**) The Lineweaver–Burk plot of recombinant SGT.

The SGT gene originated in the *S. tropica* CNB-440 strain. It belonged to glycosyltransferase family 1, comprising 1284 bp encoding a 427-amino-acid polypeptide. It was heterologously expressed in *E. coli* BL21 (DE3) after being incubated at 16 °C for nine hours with 0.3 mM IPTG (final concentration). A TALON Cobalt–Resin purification process and SDS-PAGE analysis were used to identify the crude enzyme protein. The molecular mass was estimated at about 62 kDa (Figure 1A).

### 2.2. Metabolite Biosynthesis

In the process of glycosylation, hydroxyl or functional groups of other molecules (glycosyl acceptor) are bound to a glycosyl donor. UDP-glucose and ginsenoside Rh2 were used as the donor and acceptor, respectively, in the recombinant glycosyltransferase (SGT) reaction to synthesize a new ginsenoside. The reactants were extracted using *n*-butanol, and the spot corresponding to the novel metabolite was observed on TLC (Figure 1B). This spot, however, was not seen in the control, which included SGT without Rh2, SGT and Rh2 without UDP-glucose, UDP-glucose, and UDP-glucose without Rh2 and SGT. Analyses conducted with HPLC also revealed the presence of the metabolite. (Figure 1C).

### 2.3. Kinetic Properties

Using the Michaelis–Menten model, we were able to determine the kinetics of glucosyl-ginsenoside Rh2 production by SGT. The enzyme was incubated with a non-radiolabeled UDP-glucose substrate at concentrations varying from 0.5 to 5 mM. The product was detected using HPLC to measure its kinetic parameters. The efficiency of SGT is shown in Figure 1D. According to the Lineweaver–Burk plot, the computed value for Vmax is 0.56 mol/L, min and Km is 0.012 mol/L.

### 2.4. Optimization of Temperature, pH Value, and Metal Ions in the Metabolite Synthesis

**Figure 2:**
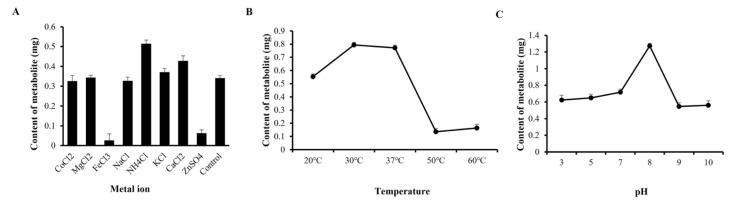
(**A**–**C**) Effect of metal ion/temperature/pH on recombinant SGT activity in synthesis of metabolite.

In comparison to the control, which contained no metal ions, the effect of metal ions on SGT revealed that enzyme activity was significantly increased by NH_4_^+^ and Ca^2+^ (Figure 2A). Furthermore, NH_4_^+^ had a considerable impact on the ideal metal ion condition for SGT. The ideal temperature, as seen in Figure 2B, was 30 °C, differing from the four other temperature levels. The best pH of SGT in 20 mM phosphate-buffered saline was pH8, which was more efficacious than other pH values (Figure 2C).

### 2.5. SGT-Mediated Metabolite Biosynthesis Pathway from Ginsenoside Rh2

For mass production, 25 mL solutions containing a combination of 2.5 mM UDP-glucose, 0.5 mM ginsenoside Rh2, and 10 mM NH_4_^+^ with SGT enzyme (pH 8, 0.1 mg/mL) were prepared. These solutions were then placed in an incubator and shaken for 12 h at 30 °C. By using open silica column chromatography and a solvent system of CHCl_3_: CH_3_OH: H_2_O (65:35:10, *v*/*v*/*v*, lower phase), the products were separated and purified. A Nova-Pak C18 column (3.9 mm 300 mm) was employed in further Repeated Waters prep HPLC analysis to accomplish separation and purification for further analysis [8]. In addition, the production of the metabolite was around 78.3% when the circumstances were optimal.

**Figure 3:**
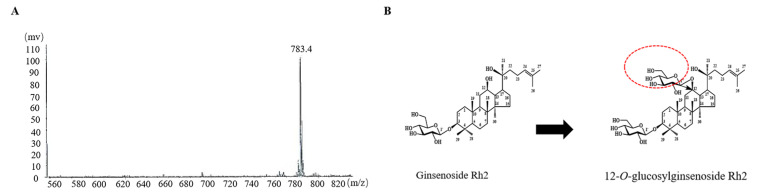
(**A**) Mass spectrum metabolite 1 after transformation by recombinant SGT. (**B**) Transformation of ginsenoside Rh2 to 12-O-glucosylginsenoside Rh2 by SGT.

The metabolite’s molecular composition is a whitish, amorphous powder, determined from the peak of the molecular ion at *m*/*z* 783.4 ([M − H]^−^, calculated for C_42_H_71_O_13_ 783.4973) (Figure 3A) in the negative high-resolution fast atom bombardment mass spectroscopy (HRFABMS) and ^13^C-NMR distortionless enhancement by polarization transfer (DEPT) spectra. The metabolite’s IR spectrum revealed bands of absorption that may be attributed to the OH group at 3401 cm^−1^ and the double bond at 1649 cm^−1^. However, except for an extra hexose sugar, the ^1^H- and ^13^C-NMR results were comparable to those of ginsenoside Rh2. The sugar’s ^13^C-NMR signals were found to be those of a hemiacetal (δ_C_ 100.7), an oxygenated methine (δ_H_ 4.51, d, J = 7.8 Hz), and an oxygenated methylene (δ_C_ 62.9), suggesting that it was *β*-glucopyranose. The anomeric proton signal’s coupling constant (δ_H_ 4.51, d, J = 7.8 Hz) was used to confirm the configuration of the anomeric hydroxy moiety. Due to the glycosylation shift, the chemical shift of C-12 (δ_C_ 79.6) was shifted downfield by 8.6 ppm in comparison to that of ginsenoside Rh2, allowing the sugar to link to the hydroxy of C-12. In addition, within the heteronuclear multiple bond correlation (HMBC) spectrum, the locations of the two sugars were determined to be C-3 and C-12, respectively. Cross-peaks were seen in the anomer proton signals H-1′ (δ_H_ 4.32, d, J = 7.8 Hz) and H-1′′ with the oxygenated methine carbon signals at δ_C_ 90.8 (C-3) and δ_C_ 79.6 (C-12), respectively. The metabolite was collectively identified as (20S)-3β,12β,20-trihydroxydammar-24-ene (3-*O*-*β*-D-glucopyranosyl)-12-*O*-*β*-D-glucopyranoside and 12-*O*-glucosylginsenoside Rh2, respectively (Figure 3B, Table S1) [34,35].

### 2.6. Anticancer Activities of the 12-O-Glucosylginsenoside Rh2 Metabolite

**Figure 4:**
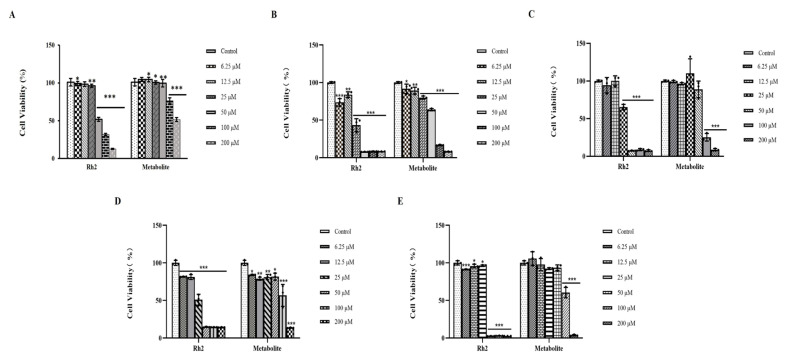
Cell viability percentage of (**A**) murine macrophage assay, (**B**) AGS proliferation assay, (**C**) HeLa proliferation assay, (**D**) U87MG proliferation assay, and (**E**) B16F10 proliferation assay of ginsenoside Rh2 and metabolite. Note: data are expressed as average ± standard error (n = 3) compared with control group, (* *p* < 0.05; ** *p* < 0.01; *** *p* < 0.001).

The pharmacological value of the novel glycosylated derivatives was tested for the treatment of cancer, and it was evaluated against ginsenoside Rh2, which is known for its antitumor effects [36]. Ginsenoside Rh2 is an anticancer agent that has been shown to be effective in treating pancreatic cancer, prostate cancer, leukemia, cutaneous squamous cell carcinoma, and glioblastoma [11,37,38,39,40]. The cell viability of ginsenoside Rh2- and 12-*O*-glucosylginsenoside Rh2-treated cancer cell lines B16F10, AGS, HeLa, and U87MG, compared to normal cell line murine macrophage RAW 274.7, were determined using the MTT colorimetric assay at a concentration range of 6.25–200 μM. According to these findings, a dose-dependent relationship exists between the novel 12-*O*-glucosylginsenoside Rh2 molecule and the reduction in the viable population of each of the four cell lines. Also, at a concentration of 200 μM, 12-*O*-glucosylginsenoside Rh2 displayed cytotoxicity against all four other cancer cell lines (Figure 4), while being less toxic with normal cell lines. Thus, the results suggest that the new derivatives have potential anticancer activity and may be considered for future pharmaceutical research. This is the first report on the activity of 12-*O*-glucosylginsenoside Rh2 against B16F10, AGS, HeLa, and U87MG cell lines.

### 2.7. Protein Structure Modeling

**Figure 5:**
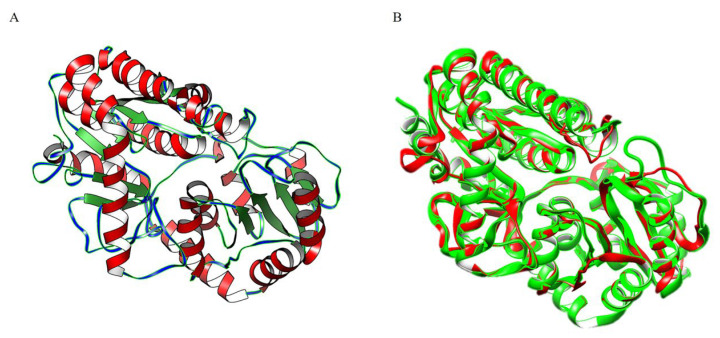
Enzyme protein structure; (**A**) protein 3D structure from I-TASSER method; (**B**) Superposition of UDPG with 5GL5. SGT protein is represented with green-colored regions, while the UGT51 protein corresponds to the red-colored regions.

When compared to the reference PDB structure of sterol 3-beta-glucosyltransferase (UGT51) from Saccharomyces cerevisiae (strain ATCC 204508/S288c), the three-dimensional structure predicted by SGT has a confidence score (C) of 0.54 and a template modelling score (TM) of 0.956 [41]. The scores for SGT and UGT51 were comparable, with a TM-score of 0.956, a root mean square deviation (RMSD) of 0.63, and an identity of 0.353%, respectively (Figure 5A,B).

### 2.8. Molecular Docking and Dynamics

**Figure 6:**
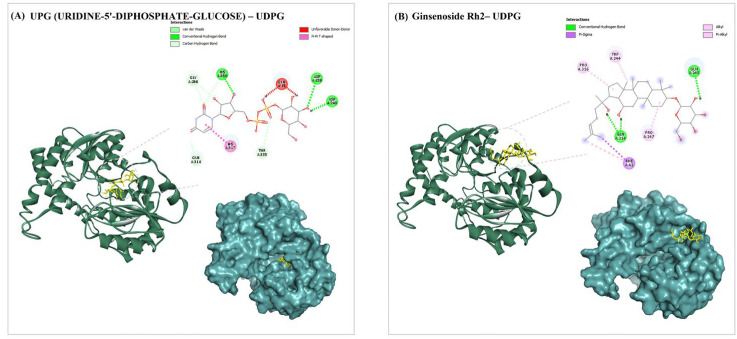
(**A**) 2D and 3D docking interactions of best-binding UPG (URIDINE-5′-DIPHOSPHATE-GLUCOSE) with UDPG. (**B**) 2D and 3D docking interactions of best-binding ginsenoside Rh2 with UDPG. Protein is depicted as a surface (cyan), and ligand is represented as sticks (yellow). Figure was generated using Discovery Studio (Dassault Systèmes BIOVIA, Discovery Studio 2023 and PyMol 2.5).

Molecular docking simulations were performed for the ginsenoside Rh2 and UDPG proteins (UDP-glycosyltransferase). In the structure of ginsenosides, modifications, such as glycosylation, usually occur at the C3 and C20-OH groups. Ginsenoside sapogenins have C-3 and/or C-20 hydroxyl groups of PPD-type, C-6 and/or C-20 hydroxyl groups of PPT-type, and OA-type saponins have C-3 hydroxyl and/or C-28 carboxyl groups [42]. The energies as well as the different amino acids interactions in the UDPG pocket are shown in Table S2. As presented in Figure 6A, UPG established hydrogen bonds and hydrophobic interactions with HIS330, ASP239, ASP240, GLY266, GLN314, THR335, HIS317, and GLN15, with a binding energy of −8.2 kcal/mol. Ginsenoside Rh2 had the highest docking score (Figure 6B). Ginsenoside Rh2 forms two hydrogen bonds with GLN314 and GLN245. The residues involved in hydrophobic interactions are PHE42, TRP244, PRO247, and PRO316, with a binding energy of −9.5 kcal/mol.

**Figure 7:**
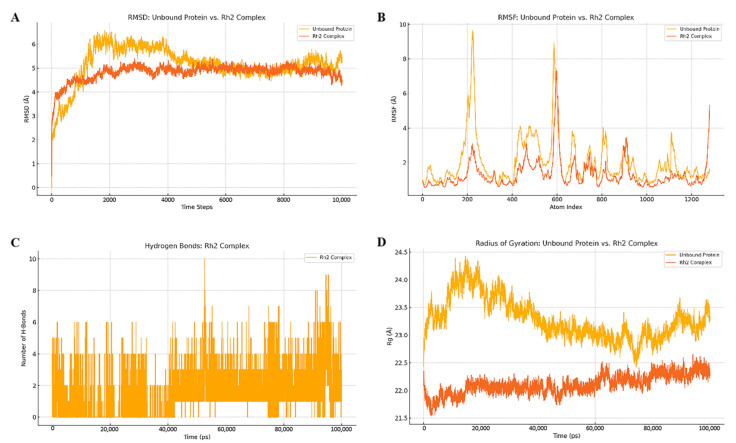
(**A**) RMSD of ginsenoside Rh2 in complex with UDPG as a function of MD simulation time (100 ns). (**B**) RMSF of ginsenoside Rh2 in complex with UDPG as a function of MD simulation time (100 ns). (**C**) Line plots of Ligand–protein H bonds for UDPG and Ginsenosides Rh2 (100 ns). (**D**) Radius of gyration plots of molecular dynamics (MD) simulation of UDPG-ginsenoside Rh2 complexes (100 ns). All axes are labeled with units in Ångström (Å).

The conformation with C20-OH or C6-OH near the catalytic residues and sugar ring of UDP-Glc was chosen and applied to the MD simulation. An all-atom MD simulation analysis was conducted, since this was thought to be an effective method for confirming the stability of the projected docked complex. The implementation of such research would also provide helpful insights on the dynamic behavior of the ligand and protein, as well as an assessment of the ligand’s principal binding contacts with important catalytic site residues [43]. The docked complexes were subjected to MD simulations in order to examine their degree of stability, interaction, and flexibility. In order to have a better understanding of the docked complexes’ stability in a biological setting, RMSD profiles were assessed for each of them. From the RMSD values depicted in Figure 7A, we are able to deduce that there was no change in the root mean square deviation of the structures that were formed over the whole course. The plot of the root mean square fluctuation (RMSF) indicates that there were only slight variations in the residues in the catalytic and binding sites of the acceptor and donor molecules (Figure 7B). Analyses were conducted on both the radius of gyration (Rg) and the number of hydrogen bonds (Figure 7C). The degrees of compactness and stability were evaluated based on the Rg values. As shown in Figure 7D, the protein structure was rather stable since the apo structure had a nearly the same Rg value between 0 and 100 ns.

In addition to molecular docking and dynamics, MM-PBSA analysis was conducted to determine the thermodynamic parameters of the UDPG-Rh2 complex, including bind-ing free energy (ΔE_binding_), van der Waals energy (ΔE_VDWAALS_), electrostatic energy (ΔE_EL_), polar solvation energy (ΔE_PB_), and non-polar solvation energy (ΔE_NPOLAR_). The results in Table S3 show that the calculated binding free energy (Δ_TOTAL_) is −22.34 kcal/mol, indicating a reasonably favorable binding interaction. This is driven by significant contributions from gas-phase interactions (ΔG_GAS_ = −140.56 kcal/mol) and solvation effects (ΔG_SOLV_ = 162.9 kcal/mol), with van der Waals (ΔE_VDWAALS_ = −60.35 kcal/mol) and electrostatic (ΔE_EL_ = −80.21 kcal/mol) interactions playing key roles. These findings highlight the thermodynamic stability of the UDPG-Rh2 complex.

## 3. Discussion

Ginseng is well known for beneficial properties in Asian countries. In particular, rare ginsenosides’ biological and therapeutic activities have been reported as cures for various cancers, obesity, diabetes, inflammation, and tumors. However, they are present at low levels in ginseng, which does not meet the requirements of pharmaceutical industries. Indeed, the low production rate and poor solubility have delayed the biological investigation and application of Rh2 and other rare ginsenosides. In consideration of these limitations, it is necessary to increase the conversion rate of rare ginsenoside and develop the synthesis of novel ginsenosides. In general, biological methods have been recommended that have advantages for the biosynthesis of minor ginsenosides, including novel ginsenosides, and which cannot produce byproducts, side reactions, and environmental pollution [44]. Moreover, structural modifications of saccharide groups of ginsenosides become effective in obtaining rare ginsenosides. In recent years, extensive research has reported that UGTs in microorganisms show higher selectivity for the glycosylation of ginsenoside in vitro [45], even though UGTs in plants are the key enzymes in the growth of the ginsenoside production pathway in vivo. In such situations, the great mass of microbial UGTs in previous studies was confirmed to glycosylate the sugar group at the C-3 position of ginsenosides, specifically [7,26].

In our study, one novel ginsenoside was successfully produced by a recombinant enzyme (SGT) expressed in *E. coli* BL21 using Rh2 as acceptor and UDPG as donor, which was identified to be glycosylated at the C-12 hydroxyl site for the first time. The purified SGT enzyme was chosen for the biocatalytic reactions. We developed an HPLC method for the assay of the metabolite after the reaction. Based on the analysis, one new product was found among the reactants. Subsequently, it was necessary to establish the hydroxyl activity of the enzyme. In order to detect the effect of SGT enzyme activity, the essential conditions of metal ions, temperature, and pH were optimized for the biosynthesis of Rh2 in vitro. According to the results, SGT exhibited glycosylation activity in the biotransformation of ginsenoside Rh2 under the values ranging from 20 to 30 °C and a pH of 3 to 10, where the best activity shown at 30 °C and pH 8. With the temperature rising from 37 to 60 °C, the repression of the SGT enzyme was clearly observed. The effect of pH value on the glycosylated reaction suggested that there was no obvious difference in the metabolite content at pH 3–7 and 9–10, whereas the maximum content was achieved at pH 8. Meanwhile, it was interesting that NH_4_^+^ played a significant role in stimulating SGT enzyme activity, compared with no metal ions. In contrast, both Fe^3+^ and Zn^2+^ showed inhibition of SGT enzyme activity. As well, the product yield of the metabolite was calculated by HPLC analysis. When conditions were optimal, the production of the metabolite achieved a maximum yield of around 78.3%. Through the HR-MS and NMR analysis, the molecular weight and structure characterization were confirmed. These results indicated that SGT displayed specificity in converting Rh2 to novel ginsenoside (20S)-3β,12β,20-trihydroxydammar-24-ene (3-*O*-β-D-glucopyranosyl)-12-*O*-β-D-glucopyranoside (12-*O*-glucosylginsenoside Rh2). The novel ginsenoside is distinguished from previous studies, in which glucose moiety is attached to the C-12 position of ginsenoside Rh2. In summary, we discovered that the bio-glycosylation role of SGT is to catalyze the C-12 hydroxyl group for glycosylation as a novel structure.

In our previous studies, we identified multiple UDP—glycosyltransferases derived from microorganisms. These enzymes selectively modified ginsenosides with UDP—glucose, resulting in the formation of novel ginsenosides that exhibited remarkable biological activity against a variety of diseases [7,30]. It is also of great significance to investigate the application of other sugar donors in glycosylation research. There is a need to explore the reactivity of sugar donors such as TDP—glucose and GDP—mannose with bioactive compounds. As far as we know, diverse sugar donors may confer unique biological activities and physicochemical properties to glycosylated products. By comparing the reaction efficiencies of different sugar donors and the characteristics of the products, the most appropriate sugar donor can be selected for specific bioactive compounds. In addition, various other natural products can serve as substrates for glycosyltransferases, including flavonoids, terpenoids, alkaloids, etc. For example, flavonoids possess multiple biological activities. However, there are deficiencies in terms of water-solubility and stability, which restrict applications. By selecting appropriate flavonoid compounds as substrates and catalyzing their glycosylation through glycosyltransferases, novel flavonoid glycoside products can be synthesized. These flavonoid glycoside products may exhibit enhanced water-solubility, improved stability, and potentially biological activities, thereby holding considerable prospects in various fields, such as pharmaceuticals, food science, and cosmetics.

In case of molecular docking and dynamics, multiple conformations were generated with better binding mechanisms and interactions inside the receptor pocket. The conformations that had values for RMSD that were the most acceptable and that had the same binding modes as the ligand were chosen. The docked complexes were subjected to MD simulations in order to examine their degree of stability, interaction, and flexibility, and it was found that the substratum was attached very strongly to the corresponding binding pockets. The number of hydrogen bonds with good bonding criteria was expressed by the hydrogen occupation during the simulation time. The hydrogen bond significantly contributes to the strong interaction between the ligand and receptor. Hydrogen bond analysis showed the strong band of the candidate ligand, which maintained more hydrogen bonds throughout the simulation. Therefore, molecular docking and dynamics analysis showed that the selected ligand formed a stable complex.

Cancer has been the second leading causes of death on a global scale [46]; stomach cancers, female cervical carcinoma, skin cancer, and glioblastoma cancer have especially attracted global attention. Unfortunately, severe adverse reactions in patients can be caused by traditional chemotherapy drugs. Thus, there is a dire need for more powerful and effective therapeutics. The ginsenoside Rh2, a well-known active ingredient in ginseng, has been established to improve the effectiveness of clinical treatment. Furthermore, numerous studies have shown that ginsenoside Rh2 is a strong inhibitor to cancer cells. Previous studies have established the mechanisms by which ginsenoside Rh2 induces apoptosis in cancer cells, revealing several fascinating pathways that contribute to its potential anticancer effects. For instance, ginsenoside Rh2 has been demonstrated to promote cell apoptosis in T-cell acute lymphocytic leukemia through the regulation of the MAPK and PI3K/AKT signaling pathways [47]. Moreover, the anticancer effects and related mechanisms of ginsenoside Rh2 in various cancer cells have been demonstrated, including its promotion of the apoptosis of related genes in cancer cells by regulating the Bax and Bcl-2 pathway, activating the mitochondrial apoptotic pathway, affecting the intracellular redox balance by the generation of ROS (reactive oxygen species), etc. Therefore, further study into the anticancer mechanisms of our novel ginsenoside is feasible, based on the existing theoretical evidence.

Herein, we evaluated the new compound and ginsenoside Rh2′s anticancer properties side by side and found that all four cancer cell lines were significantly cytotoxic to both compounds at 200 μM. Furthermore, this examination of the new molecule 12-*O*-glucosylginsenoside Rh2′s biological activities against anticancer cell lines highlights the significance of this compound as a highly effective agent for future pharmacological investigations on angiogenesis and cancer-related problems. The findings were consistent with our expectations, and confirmed the results of our previous research. On the basis of the anticancer mechanisms of Rh2, we need to demonstrate the induction of apoptosis and the inhibition of the proliferation of 12-*O*-glucosylginsenoside Rh2 in further studies. Additionally, its advantages are further demonstrated by applications in diversified diseases treated by the bioactive natural products glycosylated using glycosyltransferases. In conclusion, we strongly believe that this study offers an additional method for novel glycosylated ginsenoside development.

## 4. Materials and Methods

### 4.1. Materials and Ginsenoside Standards

The DNA genome of S. *tropica* CNB-440 was kindly provided by Professor Jae Kyung Sohng from SunMoon University in South Korea [18]. Ginsenoside Rh2 was obtained from the Ginseng Genetic Resource Bank (Yongin-si, Republic of Korea). UDP-glucose was supplied by Sigma company (St. Louis, MO, USA). Water and HPLC-grade acetonitrile were of analytical grade, from SK Chemicals (Ulsan, Republic of Korea).

### 4.2. Molecular Cloning, Expression, and Purification of Recombinant Glycosyltransferase SGT Gene from S. tropica CNB-440

Through the use of primers containing BamHI and HindIII restriction sites, the polymerase chain reaction (PCR) was able to effectively use the target gene SGT from the genomic DNA as a template: SGTF (5′-GCT GGATCC TTG ATG CGA ATT CTG GTG-3′) and SGTR (5′-AAT CCC GCC GGG CTA CGG AAGCTT AGG-3′). The function of the primer was to bind the amplified DNA fragment, which was then put into a Pgem-T-Easy vector. The PCR product was sequenced. The recombinant expression vector, pSGT, was created by subcloning, in which amplified SGT gene segments bearing BamHI and HindIII are inserted into the same site in a pET32a (+) vector.

*E. coli* BL21 (DE3) was transformed with the recombinant plasmid pSGT using the calcium chloride method, and it was then cultivated in Luria-Bertani (LB)-ampicillin medium at 37 °C to an absorbance of 0.5 at 600 nm (OD_600_). After that, protein expression was stimulated by adding isopropyl-*β*-D-thiogalactopyranoside (IPTG) for 9 h at 16 °C, at a concentration of 0.3 mM. Centrifugation was used to separate the bacterial cultures for 15 min at 4 °C (5000× *g*). After being redissolved in 20 mM sodium phosphate buffer (pH7.0), the cells were hatched at 4 °C for 5 min. The cells were broken after five cycles of sonication at 1s intervals for three minutes on an ultrasonic processor with output at 80% while lying on ice. Afterwards, the debris was eliminated by centrifuging at 12,000× *g* for 20 min at 4 °C. Using a kit from Takara Bio, Kusatsu, Shiga, Japan, purification was achieved using TALON Cobalt–Resin for His-tag purification [48,49] and then was analyzed for protein expression by SDS-PAGE (12%).

### 4.3. Biosynthesis of Metabolite

In order to evaluate glycosylation capability, an overexpressed SGT enzyme, with a final concentration in the solutions of 0.1 mg/mL and a pH of 7.0, was combined with 2.5 mM UDP-glucose and 0.5 mM Rh2. For 12 h, the mixes were incubated at 30 °C, then under the same circumstances, three groups of controls were incubated: control 1 (C1) included SGT; control 2 (C2) contained SGT and UDP-glucose; control 3 (C3) contained UDP-glucose; and control 4 (C4) contained ginsenoside Rh2 and UDP-glucose.

### 4.4. Enzyme Kinetic Properties

The Michaelis–Menten constant, the kinetic parameters, Vmax (mol/L·min), and the Km (mol/L), using various ratios of UDP-glucose ranging from 0.5 mM to 5 mM, were estimated using the Michaelis–Menten plot [48]. The reaction mixtures were in a sodium phosphate buffer with a pH of 7.0 at 30 °C. Product analysis was conducted using the HPLC technique. The Lineweaver–Burk plot was used to calculate the values of Km and Vmax.

### 4.5. Optimization of Effect of SGT Activity

In the first synthesis test, 1 mL of reaction mix containing 2.5 mM UDP-glucose and 0.5 mM ginsenoside Rh2 were utilized. The study of metal ions was put to the test in the presence of Cobalt (2+), Magnesium (2+), Ferric (3+), Sodium (1+), Copper (2+), Ammonium (NH4^+^), Potassium (1+), Calcium (2+), and Zinc (2+), at a final concentration of 10 mM, with a control without metal ions for comparison. The effect of temperature of SGT enzymatic activity was investigated at 20–60 °C using sodium phosphate buffer solution (pH 7.0), under the optimal conditions for the metal ions. Similar to this, the effect of pH on SGT enzyme activity was examined following incubation at the correct temperature and metal ion concentrations in buffers with 20 mM glycine-HCl buffer at pH 3.0, citric acid–sodium citrate buffer at pH 4.0–5.0, sodium phosphate buffer at pH 6.0–7.0, Tris-HCl buffer at pH 8.0–9.0, and glycine–NaOH buffer at pH 10.0. Following the termination of the reaction by adding an equal volume of *n*-butanol that had been saturated with water in order to conduct TLC, HPLC, and HR/MS analysis (high-resolution mass spectrometry), the *n*-butanol content from each sample was allowed to evaporate until completely dry, and then was dissolved in HPLC methanol.

### 4.6. Detection Methods of Metabolite

Thin layer chromatography (TLC) examination was carried out using silica gel plates (60F254, 20 cm × 20 cm, 0.2 mm layer thickness, Merck, Darmstadt, Germany) and a developing solvent composed of methanol, chloroform, and water in the proportions of 35:65:10, respectively. The spots were identified on the TLC plate after spraying them with 10% (*v*/*v*) H_2_SO_4_ and then heating them for one minute at 110 °C [26]. Following the extraction of the reaction mixture in H_2_O-saturated *n*-butanol, it was evaporated in a vacuum. For High Pressure Liquid Chromatography (HPLC) examination, methanol was used to dissolve the remaining residue. The Agilent 1260 system (Agilent, Santa Clara, CA, USA) was used to conduct the experiment. On a C18 column that measured 250 by 4.6 mm and had a particle size of 5 μm, the separation was carried out with acetonitrile (solvent A) and distilled water (solvent B) serving as the mobile phases at the following conditions: 85% B for 5 min, 79% B for 20 min, 42% B for 55 min, 10% B for 12 min, and 85% B for 18 min, at a flow rate of 1.6 milliliters per minute. UV (203 nm) absorbance was used to detect the sample [26]. A Finnigan LCQ-Advantage mass spectrometer (Ion Mode: FAB-; Elements: C 43/0, H 72/0, O 14/0; Mass Tolerance: 1000 ppm, 10 mMu if *m*/*z* 10, 20 mMu if *m*/*z* > 20; Unsaturation: −0.5–50.0) was used to conduct the MS analysis. Using hydrogen-1 and carbon-13 nuclear magnetic resonance (NMR), the structure of the metabolite was identified. In order to collect the ^1^H-NMR and ^13^C-NMR spectra, a Bruker Av 600NMR spectrometer (Billerica, MA, USA) operating at 100 MHz was used, with pyridine-d5 as the solvent.

### 4.7. Cytotoxicity Evaluation of Metabolite with Rh2

Using the cancer cell lines of skin melanoma B16F10, gastric carcinoma AGS, Hela cervical cancer cells, and brain glioblastoma U87MG, researchers examined the effect of the metabolite on the viability and proliferation of the cancer cells. The DMEM medium (Invitrogen, Grand Island, NY, USA), containing fetal bovine serum (10%), was used to cultivate the cells (FBS, Invitrogen). In an incubator with 5% CO_2_ humidity, all cells were kept at a constant 37 °C. In 96-well plates (SPL Life Sciences, Pocheon-si, Gyeonggi, Korea), cells were seeded at a density of 2000 cells per well, and then they were treated with metabolite across concentrations. (6.25, 12.5, 25, 50, 100, 200 μM) for 72 h for the cell growth test. Cell growth was closely examined using the colorimetric 3-(4,5-dimethylthiazol-2-yl)-2,5-diphenyltetrazolium bromide (MTT) test.

### 4.8. Protein Structure Modeling, Molecular Docking, and Dynamics Simulation

#### 4.8.1. Protein Structure Modeling

The UDP-glucose sterol glucosyltransferases’ (SGTs’) three-dimensional protein structure was modeled using the I-TASSER method [50], which is one of the best protein model prediction servers in the Critical Assessment of protein Structure Prediction (CASP). The best model was selected from the I-TASSER server based on the C-score, and the structure was aligned with the reference protein structure of UGT51 with the TM-Align score [51].

#### 4.8.2. Molecular Docking and Dynamics

In this study, the binding energy and interaction of ginsenoside Rh2 with UDPG is investigated. For the molecular docking of Rh2 on the modeled UDPG protein (Figure 4A,B), the Spatial Data File (SDF) for ginsenoside Rh2 was obtained via the PubChem database (https://pubchem.ncbi.nlm.nih.gov/, accessed on 15 May 2024). It was converted to PDB format using the Open Babel program 3.1, and the structured configurations were saved in PDBQT format. The virtual testing of substances prepared as ligands against prepared protein receptors was conducted using AutoDock 4.2 [52]. Using AutoDock Tools, the basic protein structure was modified to retain the protein’s original charge and create the PDBQT file for docking. The receptor preparation was accomplished using Auto-Dock 4.2. Each protein chain’s composition was determined by eliminating solvent molecules and receptor-associated binding compounds. The edit toolbar was used to add polar hydrogens and charges, after which the receptor was saved in PDBQT format. The charges for the ligand were assigned using the AutoDock GUI, and the number of rotatable bonds and angles for the ligand were handled automatically by the docking software. The docking axis was defined based on specific coordinates, and no restraints were applied to the protein during docking. The number of docking times was adjusted to 100, and the default value for each of the other parameters was used instead. The best-scoring location was estimated using visual analysis and the docking score. Further, the docked complex was utilized for molecular dynamics simulation.

The MD studies of Ginsenoside Rh2 for the UDPG protein were performed for a period of 100 ns by using GROMACS 2020.1 software [53]. The CHARMM force field was used for all simulations, and hydrogen atoms were added to the ligand using Biovia Discovery Studio 2023. The ligand topology was generated using SwissParam (version 3.1, 2024), and its parameters were incorporated into the GROMACS topology file. A periodic cubic solvated box was constructed for the receptor–ligand complexes to reside in, and explicit water molecules (SPC216) were added. The size of the simulation box was set to a dodecahedron with a buffer distance of 1.0 nm. The system was neutralized by adding Na⁺ and Cl⁻ ions at physiological concentrations. Energy minimization and system equilibration were carried out using the NVT and NPT ensembles before executing the actual dynamics. Positional restraints were applied to the ligand during equilibration with a force constant of 1000 kJ/mol/nm to stabilize the system. Subsequently, a 100 ns molecular dynamics simulation was conducted for all molecules. The MD trajectories were analyzed using GROMACS tools to calculate root mean square deviation (RMSD), root mean square fluctuation (RMSF), radius of gyration (Rg), and the number of hydrogen bonds. The results demonstrated the stability of the enzyme–ligand complex, and one 100 ns simulation was performed for each system. MMPBSA analysis was performed using the g_mmpbsa tool in GROMACS 2020.1. The MD trajectories from the 100 ns simulations were used as input, and binding free energies were computed for the UDPG-ginsenoside Rh2 complex. The contributions of van der Waals, electrostatic, polar solvation, and non-polar solvation energies were evaluated for snapshots taken at regular intervals throughout the trajectory.

### 4.9. Statistical Analysis

All studies were independently carried out in triplicate, and the results are provided as biological duplicates in order to evaluate the significance level. The Student’s *t*-test was used to compare the mean values of the treatment group to those of the control group. Differences in the statistics that had a *p*-value of 0.05 or below were considered to be statistically significant.

## 5. Conclusions

In the current investigation, CNB-440, a gene originating from *S. tropica*, was effectively cloned, expressed heterologously in *E. coli*, and we also isolated the purified enzyme for in vitro biotransformation. The enzymatic process was optimized with NH_4_^+^ metal ions at 30 °C and pH 8.0. Additionally, our findings demonstrate a straightforward strategy for the application of engineered enzymes in metabolite biosynthesis. Through optimizing the biological synthesis method, we were able to boost the production of the novel glycosylated derivative 12-*O*-glucosylginsenoside Rh2, where glucose is attached at the C-12 position of ginsenoside Rh2, as identified by the NMR analysis. It was the end product of an in vitro process in which the flexible SGT enzyme transferred UDP-glucose, serving as a substrate, to ginsenoside Rh2, acting as an acceptor. Numerous studies have shown that ginsenoside Rh2 has been a strong inhibitor to cancer cells. We evaluated the new compound and its anticancer properties side by side, and all four cancer cell lines were significantly cytotoxic to both compounds at 200 μM. Furthermore, computational studies, including molecular docking, dynamics simulations, and MMPBSA analysis, provided critical insights into the intermolecular interactions, stability, and binding energetics of the enzyme–ligand complex. These computational findings complement the experimental data, enhancing our understanding of the structural basis of glycosylation by SGT and its functional implications. This examination of the new molecule 12-*O*-glucosylginsenoside Rh2’s biological activities against anticancer cell lines highlights the significance of this compound as a highly effective agent for future pharmacological investigations on angiogenesis and cancer-related problems. We strongly believe that this study offers an additional method for novel glycosylated ginsenoside development.

## Figures and Tables

**Table 1:** List of recent achievements in production of ginsenosides by glycosyltransferases from microorganism.

	Glycosyltransferase	Sugar Acceptor	Product	References
*Bacillus subtilis 168*	BS-YjiC	PPD	F12, Rh2	[19]
*Saccharomyces cerevisiae*	UGT1 and GTK1	PPD	CK, F2	[20]
*Bacillus subtilis*	Bs-YjiC	PPD	Rh2	[21]
*Lactobacillus rhamnosus*	LRGT	Rh2	Glucosyl ginsenoside Rh2, Diglucosyl ginsenoside Rh2	[7]
*Bacillus subtilis 168*	Bs-YjiC mutant M315F	PPD	Rh2	[22]
*Bacillus subtilis 168*	UGT-YjiC	PPD	Rh2	[23]
*Bacillus subtilis 168*	Bs-YjiC	Rg3	Rd12	[24]
*Yarrowia lipolytica*	UGT1	PPD	CK	[25]
*Saccharomyces cerevisiae*	UGTPg45	PPD	Rh2	[26]
*Bacillus subtilis*	GTC1, GTK1	PPD	CK, Rh2, F2	[27]
*Bacillus subtilis*	UGT109A1	Re, Rf, Rh1 and R1	3,20-di-*O*-β-_D_-glucopyranosyl-6-*O*-[α-L-rhamnopyrano-(1→2)-β-D-glucopyranosyl]-dammar-24-ene-3β,6α,12β,20S-tetraol, 3,20-di-*O*-β-D-glucopyranosyl-6-*O*-[β-D-xylopyranosyl-(1→2)-β-Dglucopyranosyl]-dammar-24-ene-3β,6α,12β,20S-tetraol, 3-*O*-β-D-glucopyranosyl-6-*O*-[β-Dglucopyranosyl-(1→2)-β-D-glucopyranosyl]-dammar-24-ene-3β,6α,12β,20S-tetraol, 3,12-di-*O*-β-Dglucopyranosyl-6-*O*-[β-D-glucopyranosyl-(1→2)-β-D-glucopyranosyl]-dammar-24-ene-3β,6a,12β,20S-tetraol, 3,6-di-*O*-β-D-glucopyranosyl-dammar-24-ene-3β,6α,12β,20S-tetraol, 3,6,12-tri-*O*-β-D-glucopyranosyl-dammar-24-ene-3β,6α,12β,20S-tetraol	[28]
*Bacillus subtilis 168*	Bs-YjiC	PPD	F12	[29]
*Bacillus subtilis*	BSGT1	F1	Ia	[30]
*Bacillus subtilis 168*	Bs-YjiC	PPT	Rh1	[29]
*Bacillus subtilis*	UGT109A1	DM, PPD, PPT	3β-*O*-Glc-DM and 3β,20S-Di-*O*-Glc-DM, 3β,12β-Di-*O*-Glc-PPD, 3β,12β-Di-*O*-Glc-PPT	[31]
*Saccharomyces cerevisiae*	UGT51	PPD	Rh2	[32]
*Saccharomyces cerevisiae*	UGTPg45, UGTPg29	PPD	Rh2, Rg3	[33]

## Data Availability

All analyzed data generated in this study are available within the article.

## Supplementary Materials

The following supporting information can be downloaded at: https://www.mdpi.com/article/10.3390/molecules30040898/s1, Table S1. 1H- (600 MHz) and 13C-NMR (150 MHz) spectra of the 12-O-glucosylginsenoside Rh2. Table S2. Interaction of Ginsenoside Rh2 and UPG with amino acid residue of UDPG. Table S3. Calculated Binding Free Energy (MMPBSA) for UDPG-Rh2 Complex Post MD Simulation. Figure S1. HRFABMS spectrum of metabolite 1. Figure S2. ^1^H NMR spectra of metabolite 1. Figure S3. ^13^C NMR spectra of metabolite 1. Figure S4. COSY spectra of metabolite 1. Figure S5. Edited HSQC spectra of metabolite 1. Figure S6. HMBC spectra of metabolite 1. Figure S7. DEPT45 spectra of metabolite 1. Figure S8. DEPT90 spectra of metabolite 1.
