# Supplementary material for: Biosynthesis of a Novel Ginsenoside with High Anticancer Activity by Recombinant UDP-Glycosyltransferase and Characterization of Its Biological Properties
Source: Molecules. 2025 Feb 14;30(4):898. doi: 10.3390/molecules30040898 (PMC11858633; doi:10.3390/molecules30040898)
Supplement: Supplementary file 1 [file molecules-30-00898-s001.zip › Supplementary Figures.pptx]

## Slide 1
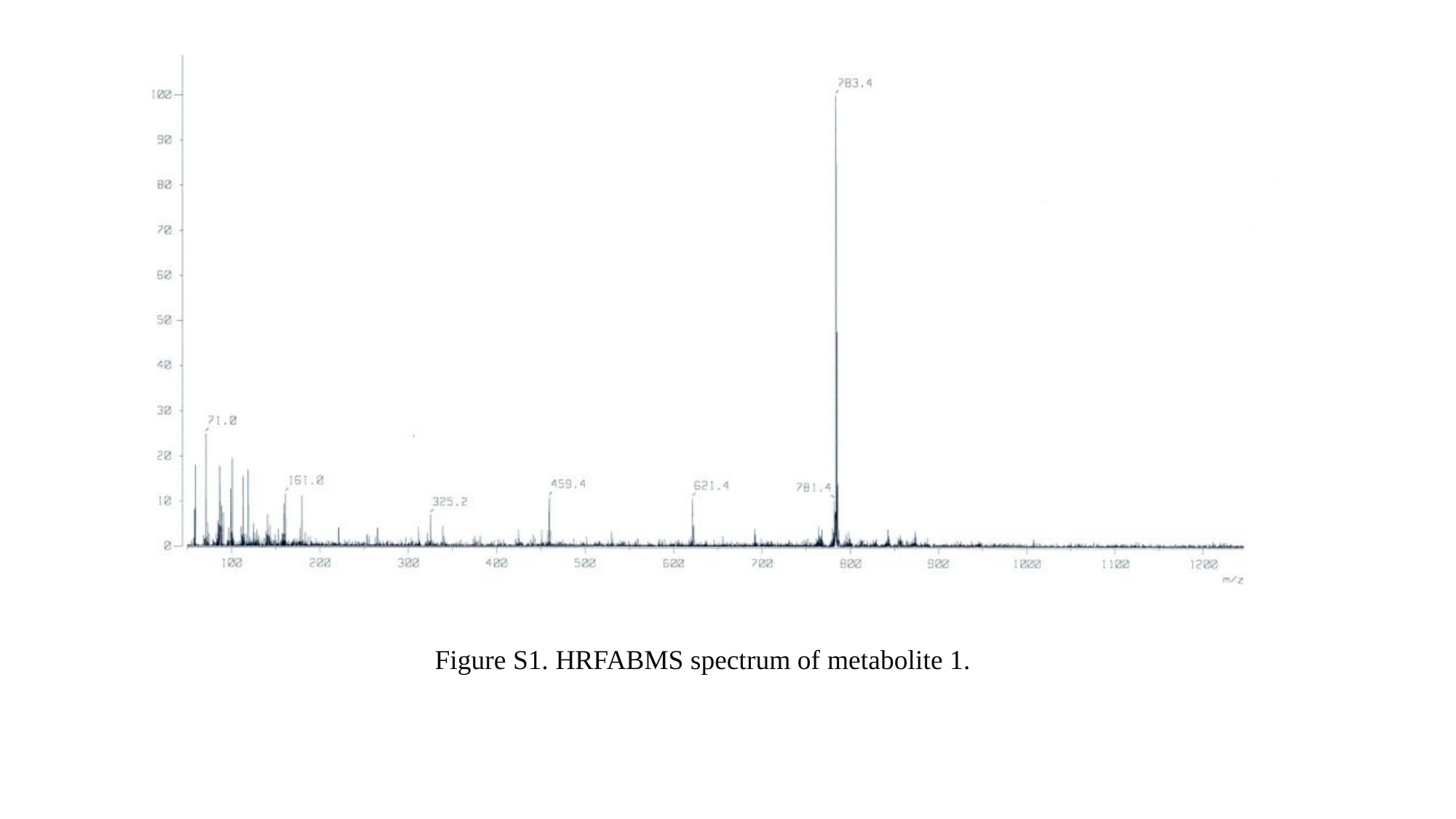

Figure S1. HRFABMS spectrum of metabolite 1.

## Slide 2
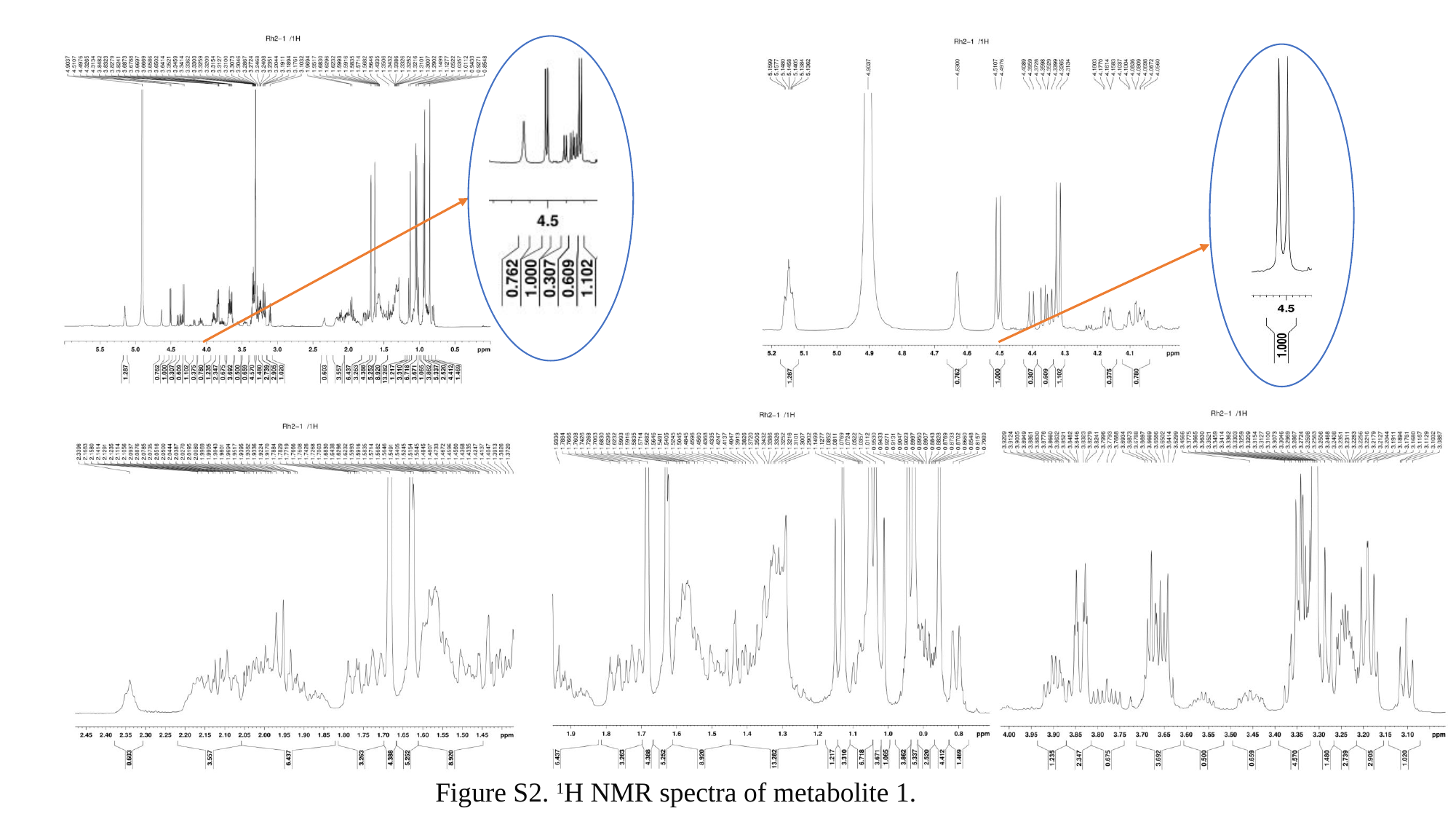

Figure S2. 1H NMR spectra of metabolite 1.

## Slide 3
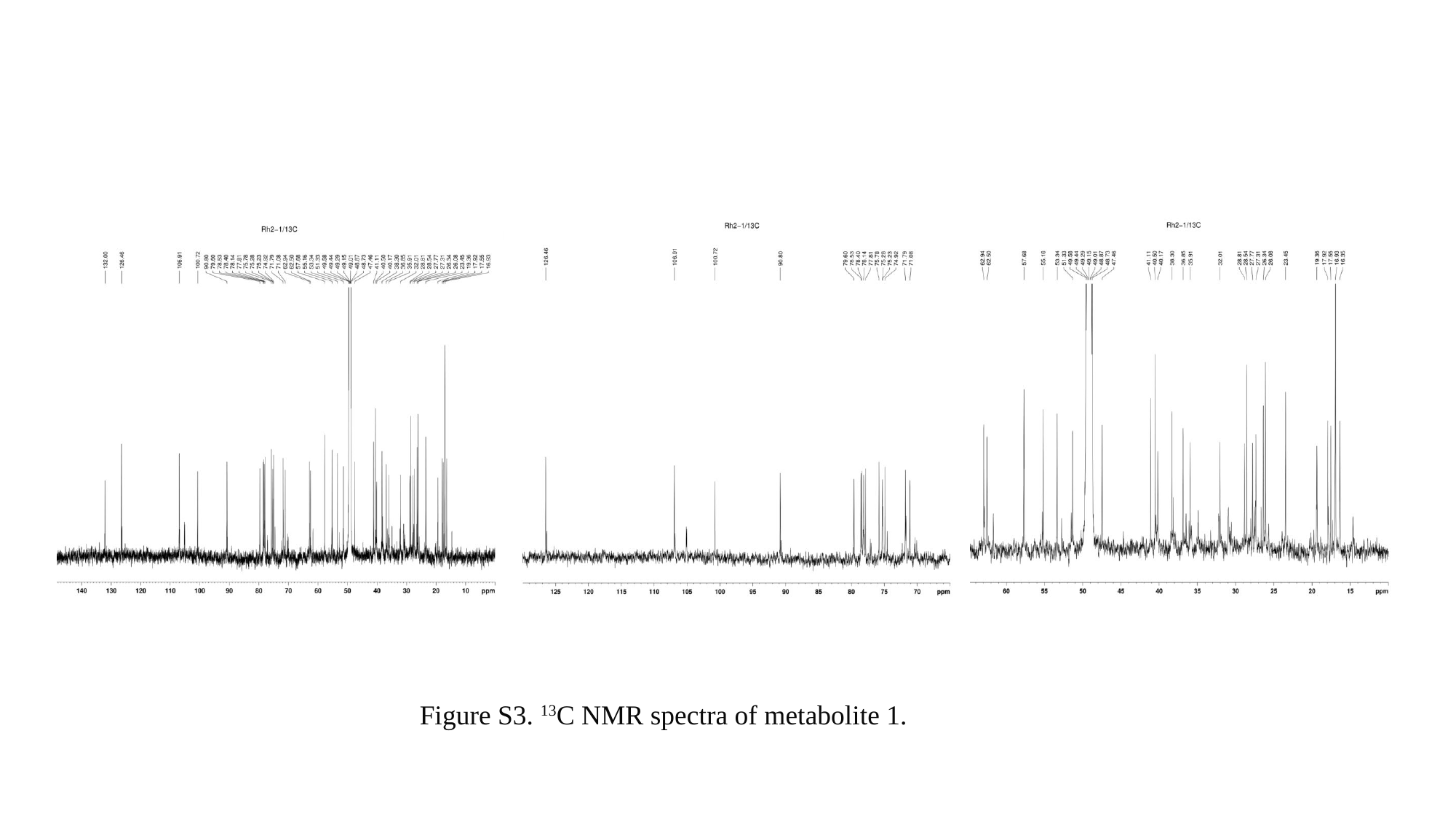

Figure S3. 13C NMR spectra of metabolite 1.

## Slide 4
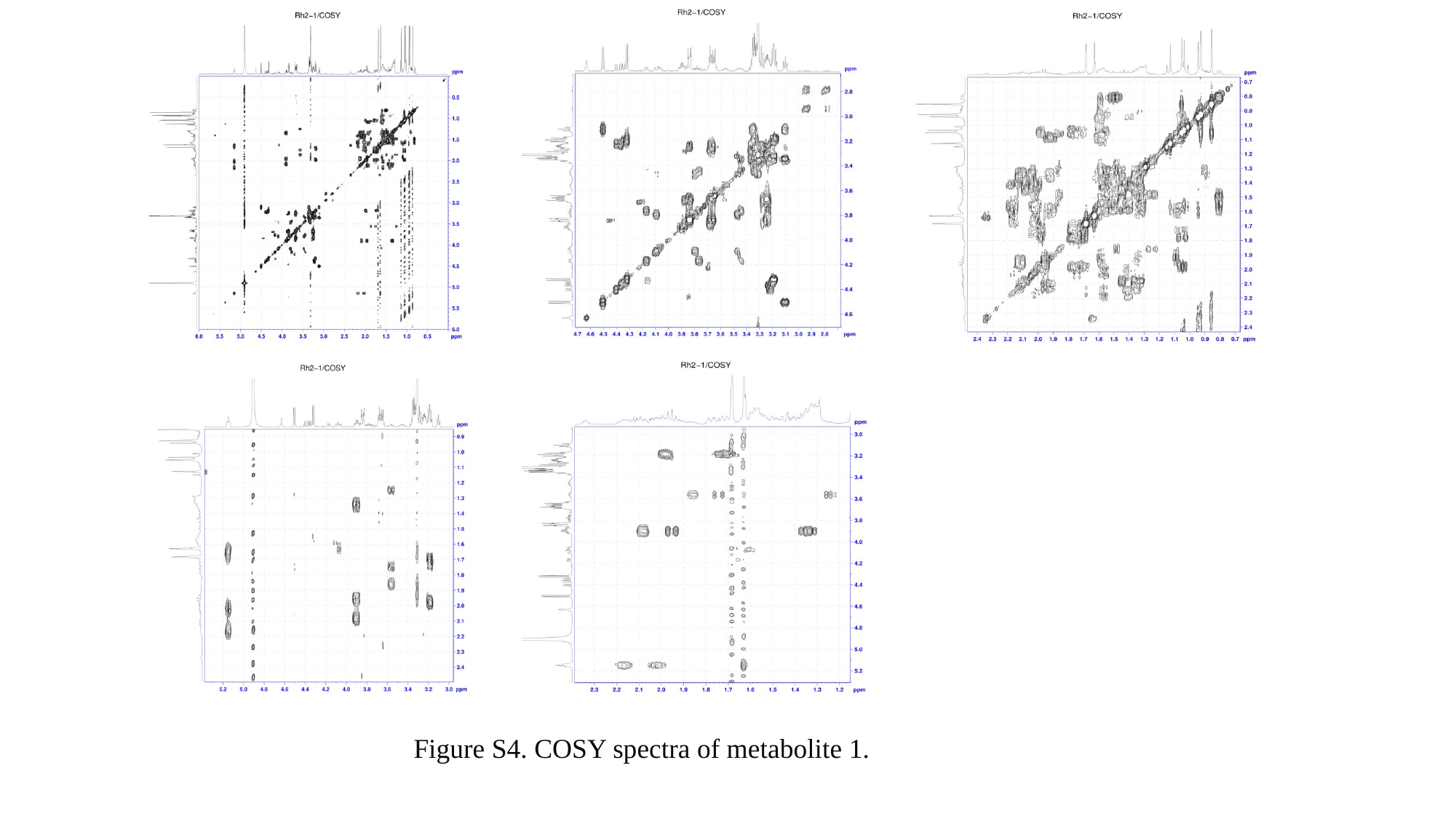

Figure S4. COSY spectra of metabolite 1.

## Slide 5
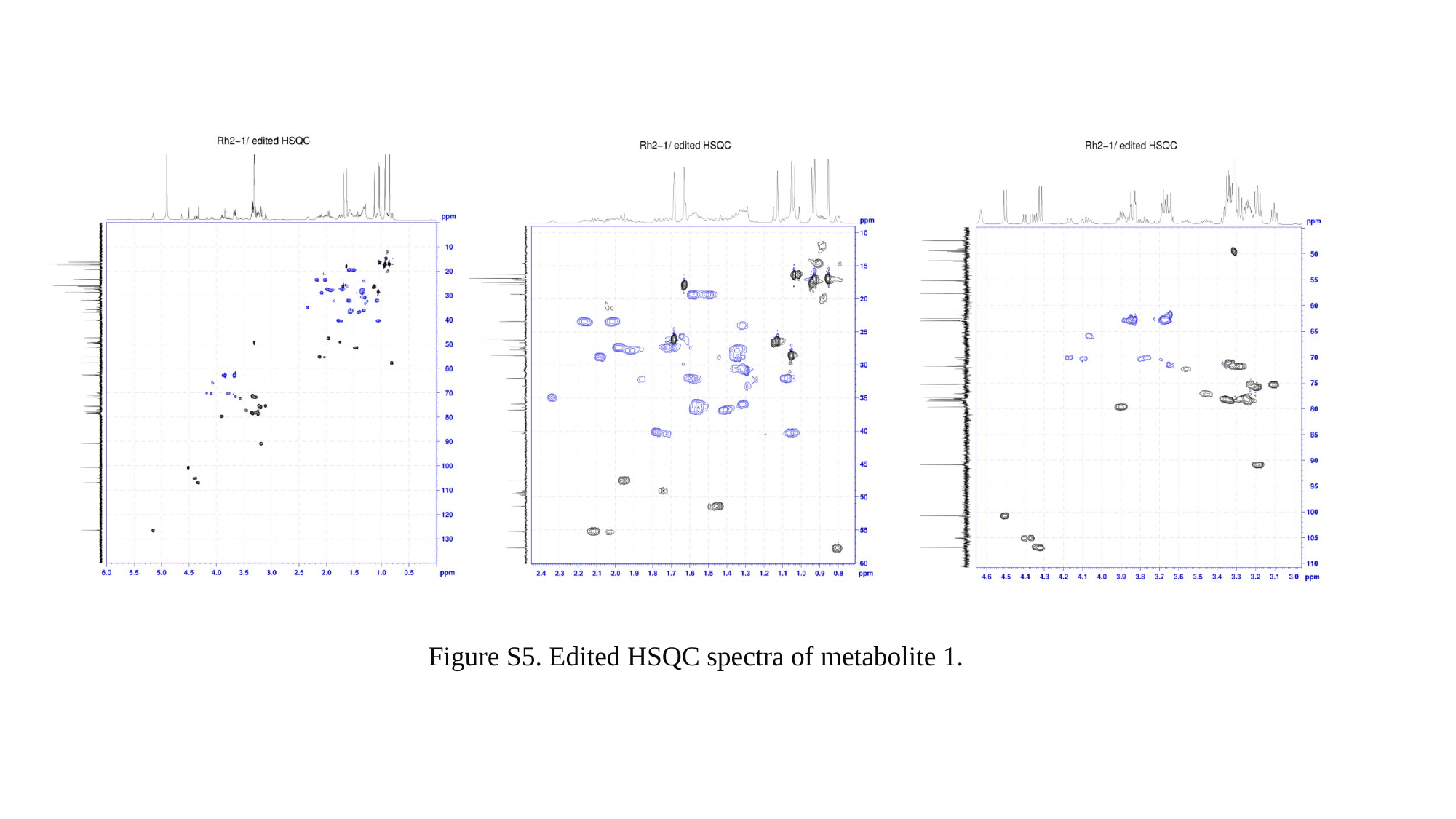

Figure S5. Edited HSQC spectra of metabolite 1.

## Slide 6
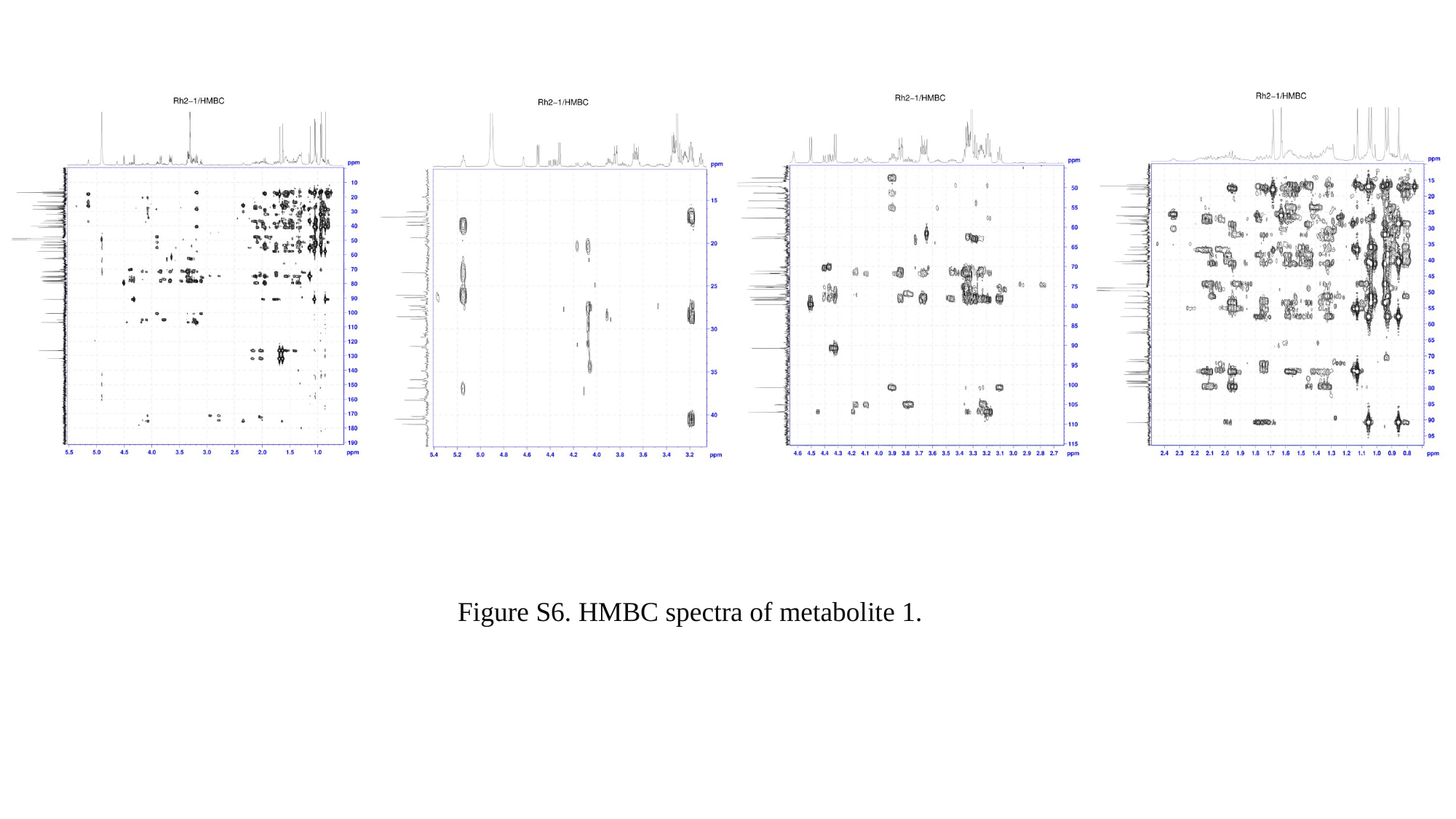

Figure S6. HMBC spectra of metabolite 1.

## Slide 7
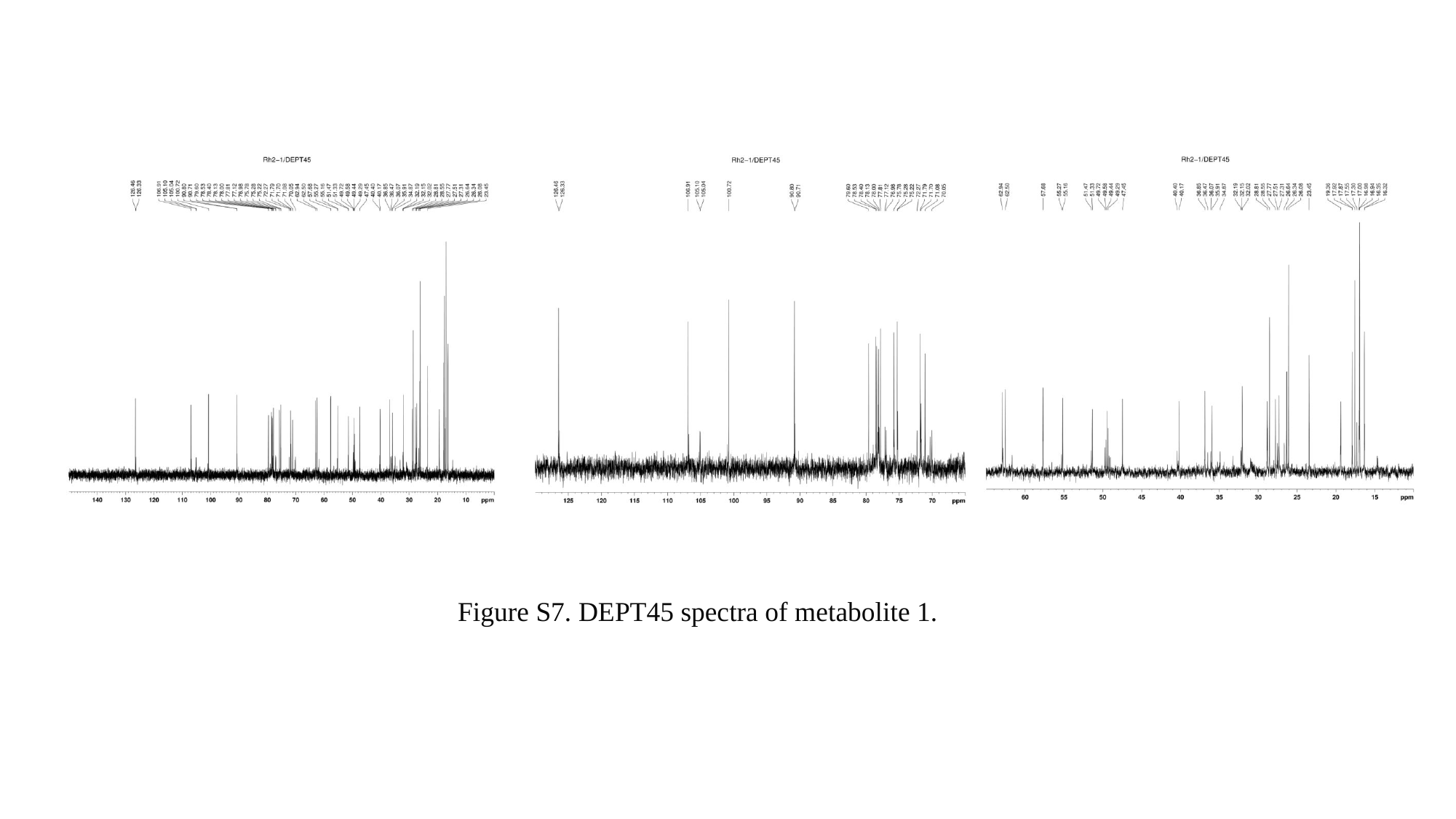

Figure S7. DEPT45 spectra of metabolite 1.

## Slide 8
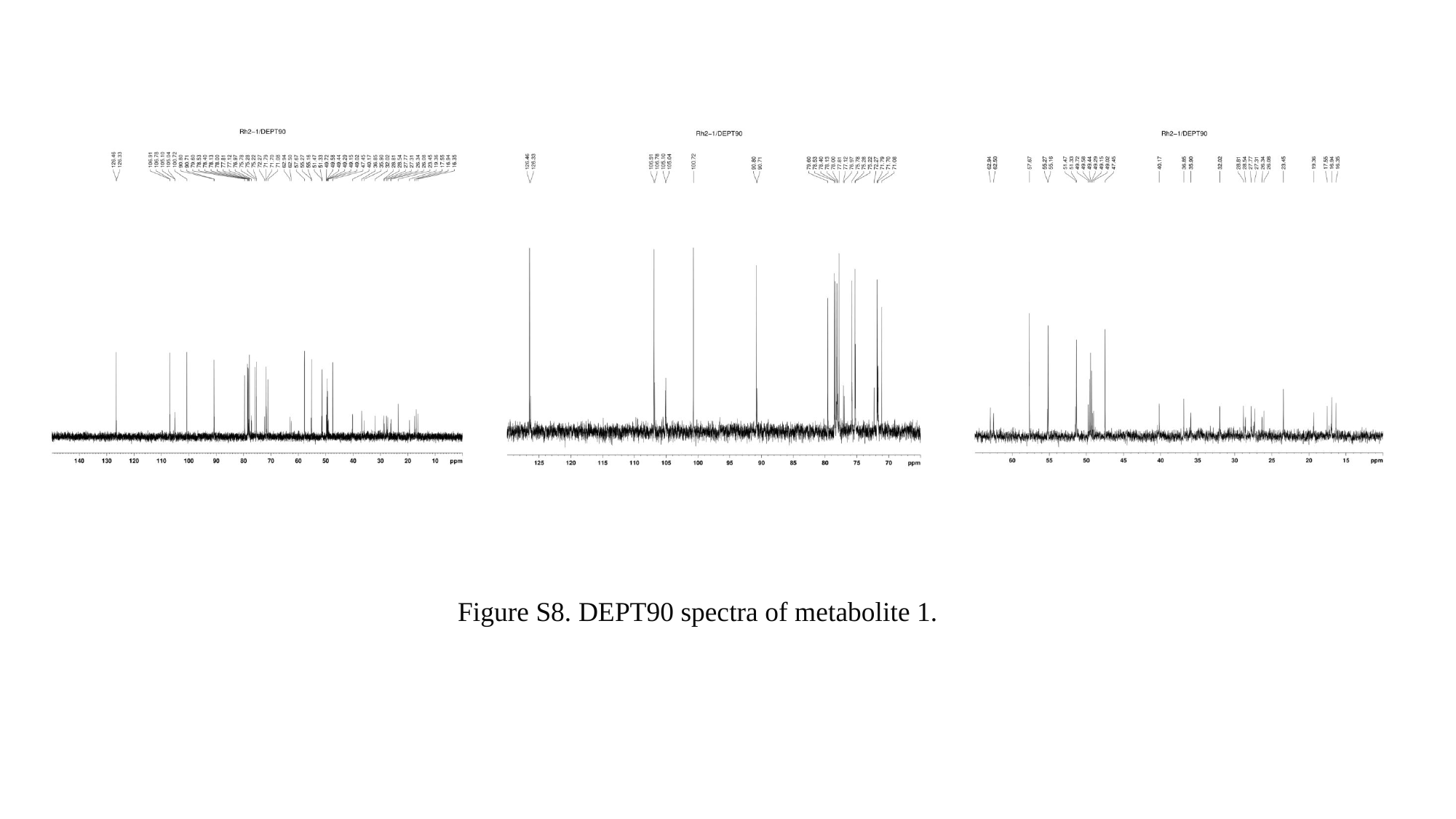

Figure S8. DEPT90 spectra of metabolite 1.

## Slide 9
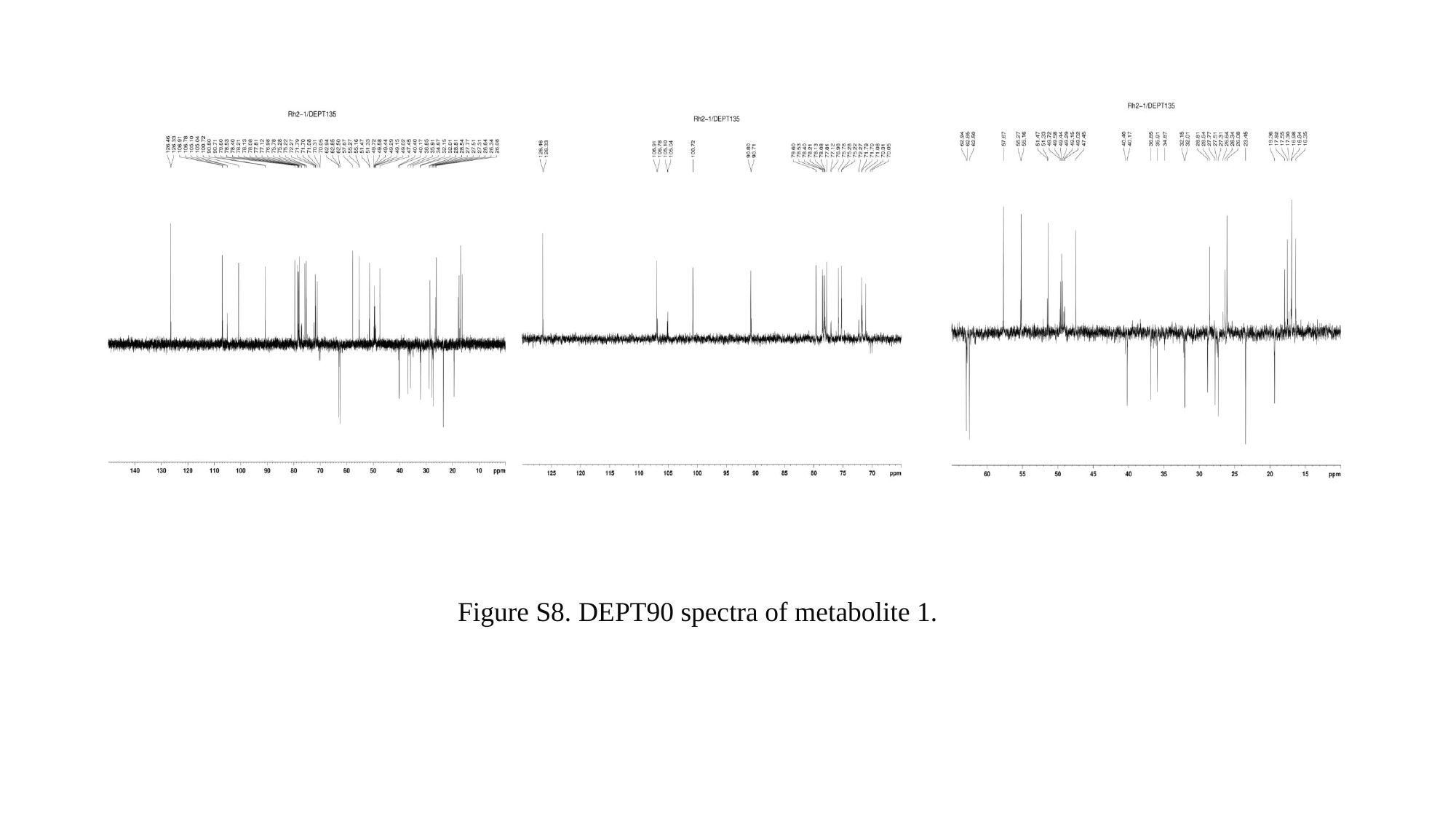

Figure S8. DEPT90 spectra of metabolite 1.
