# Supplementary material for: Biosynthesis of a Novel Ginsenoside with High Anticancer Activity by Recombinant UDP-Glycosyltransferase and Characterization of Its Biological Properties
Source: Molecules. 2025 Feb 14;30(4):898. doi: 10.3390/molecules30040898 (PMC11858633; doi:10.3390/molecules30040898)
Supplement: Supplementary file 1 [file molecules-30-00898-s001.zip › Supplementary Tables.pdf]

**Table S1.** 1H- (600 MHz) and 13C-NMR (150 MHz) spectra of the 12-O-glucosylginsenoside Rh2.

| No of C | δC | δH |
|---|---|---|
| 1 | 40.5 | 1.72, 1.09, overlapped |
| 2 | 27.3 | 1.97, 1.58, m |
| 3 | 90.8 | 3.19, overlapped |
| 4 | 40.2 | - |
| 5 | 57.7 | 0.81, br d, J=10.1 Hz |
| 6 | 19.4 | 1.58, 1.49, overlapped |
| 7 | 35.9 | 1.54, 1.32, overlapped |
| 8 | 41.1 | - |
| 9 | 47.5 | 1.48, overlapped |
| 10 | 38.3 | - |
| 11 | 32.0 | 1.39, 1.31, m |
| 12 | 79.6 | 3.90, ddd, J=10.4, 10.4, 5.1 Hz |
| 13 | 55.2 | 1.93, m |
| 14 | 51.3 | - |
| 15 | 28.8 | 1.88, 1.59, overlapped |
| 16 | 27.8 | 1.96, 1.37, overlapped |
| 17 | 53.3 | 2.08, m |
| 18 | 16.9 | 1.03, s |
| 19 | 17.6 | 0.94, s |
| 20 | 74.9 | - |
| 21 | 26.3 | 1.12, s |
| 22 | 36.9 | 1.57, 1.43, overlapped |
| 23 | 23.5 | 2.15, 2.02, m |
| 24 | 126.5 | 5.14, dd, J=8.4, 7.1 Hz |
| 25 | 132.0 | - |
| 26 | 26.1 | 1.69, s |
| 27 | 17.9 | 1.64, s |
| 28 | 28.5 | 1.05, s |
| 29 | 16.4 | 0.85, s |
| 30 | 16.9 | 0.92, s |
| C-1' | 106.9 | 4.32, d, J=7.8 Hz |
| C-2' | 75.8 | 3.17, overlapped |
| C-3' | 78.5 | 3.33, overlapped |
| C-4' | 71.8 | 3.35, overlapped |
| C-5' | 78.4 | 3.23, m |
| C-6' | 62.5 | 3.85, dd, J=12.3, 2.2 Hz, 3.67, dd, J=12.3, 5.1 Hz |
| C-1'' | 100.7 | 4.51, d, J=7.8 Hz |
| C-2'' | 75.3 | 3.10, dd, J=8.1, 8.1 Hz |
| C-3'' | 78.1 | 3.34, overlapped |
| C-4'' | 71.1 | 3.29, overlapped |
| C-5'' | 77.8 | 3.24, m |
| C-6'' | 62.9 | 3.85, dd, J=12.3, 2.4 Hz, 3.65, dd, J=12.3, 5.3 Hz |

**Table S2.** Interaction of Ginsenoside Rh2 and UPG with amino acid residue of UDPG.

| Protein | Compound | Binding Energy (kcal/mol) | Hydrogen Bond Interactions | Other Interactions | No. of Hydrogen Bonds |
|---|---|---|---|---|---|
| UDPG | UPG | -8.2 | HIS330, ASP239, ASP240 | GLY266, GLN314, THR335, HIS317, GLN15 | 3 |
| | Ginsenoside Rh2 | -9.5 | GLN314, GLN245 | PHE42, TRP244, PRO247, PRO316 | 2 |

**Table S3.** Calculated Binding Free Energy (MMPBSA) for UDPG-Rh2 Complex Post MD Simulation.

| Component | UDPG-Rh2 Complex |
|---|---|
| $\Delta E_{VDWAALS}$ (kcal/mol) | -60.35 |
| $\Delta E_{EL}$ (kcal/mol) | -80.21 |
| $\Delta E_{PB}$ (kcal/mol) | 112.78 |
| $\Delta E_{NPOLAR}$ (kcal/mol) | -50.12 |
| $\Delta E_{DISPER}$ (kcal/mol) | 95.43 |
| $\Delta G_{GAS}$ (kcal/mol) | -140.56 |
| $\Delta G_{SOLV}$ (kcal/mol) | 162.9 |
| $\Delta_{TOTAL}$ (kcal/mol) | -22.34 |
